# A Case of Persistent Bilateral Irvine-Gass Syndrome Following Uneventful Cataract Surgery in a Healthy Individual

**DOI:** 10.7759/cureus.85512

**Published:** 2025-06-07

**Authors:** Chin Shin Low, Teck Chee Cheng, Jemaima Che Hamzah, Rona Asnida Nasaruddin

**Affiliations:** 1 Ophthalmology, Universiti Kebangsaan Malaysia Medical Centre, Kuala Lumpur, MYS

**Keywords:** cataract, intravitreal dexamethasone implant, irvine-gass syndrome, orbital floor triamcinolone injection, pseudophakic cystoid macular edema

## Abstract

Pseudophakic cystoid macular edema (PCME), also known as Irvine-Gass Syndrome, is one of the most common causes of postoperative visual impairment, which is usually self-limiting in nature. We report a case of persistent bilateral Irvine-Gass Syndrome (IGS) up to 11 months in a healthy individual despite an uneventful cataract surgery. A 58-year-old e-hailing male driver without any underlying systemic comorbidities complained of cloudy vision six weeks after uneventful bilateral cataract surgery. His best corrected visual acuity (BCVA) measured with the Early Treatment Diabetic Retinopathy Study (ETDRS) chart was 6/15 OD and 6/18 OS, which were initially thought to be attributed to bilateral early-onset posterior capsule opacity. At six months postoperatively, he underwent neodymium:yttrium-aluminum-garnet (Nd:YAG) posterior capsulotomy for both eyes. BCVA slightly improved to 6/12 OU, but optical coherence tomography (OCT) of both maculae showed cystoid macular edema. Initial treatment with topical nepafenac 0.1% three times daily was prescribed for three months, but Irvine-Gass Syndrome persisted; hence, bilateral orbital floor triamcinolone (OFTA) of 40 mg/1 ml injection was given. Anti-vascular endothelial growth factor (VEGF) was not considered in this case due to cost factors. Post orbital floor triamcinolone injection, there was persistent intraretinal cystic fluid. Unfortunately, there was no gain in visual acuity due to residual cystoid macular edema. Treatment was further escalated to sustained-release intravitreal dexamethasone implant 0.7 mg for both eyes. Following the dexamethasone implant, the bilateral macular edema resolved and was maintained throughout 120 days. This case illustrates the stepwise treatment approach in this patient without the presence of a definitive management guideline.

## Introduction

Irvine-Gass syndrome (IGS) is a condition also known as pseudophakic cystoid macular edema (PCME), which is a common cause of painless postoperative visual decline. Despite advances in this era of modern ophthalmology, where small incision cataract surgery (SICS) and phacoemulsification are designed to revolutionize cataract surgery and minimize the risks of developing pseudophakic cystoid macular edema, this complication remains unavoidable due to the sheer volume of cataract surgeries performed worldwide. The incidence of PCME is about 0.1-2.4% following an uneventful cataract surgery [1]. The highest incidence was recorded at the five-week postoperative interval [2].

The diagnosis of PCME can be achieved through clinical examination, optical coherence tomography, or angiographic examination, and its incidence varies accordingly. Of these three techniques, optical coherence tomography (OCT) is the most superior in sensitivity, but angiography remains the gold standard because of its ability to delineate causes of cystoid macular edema [3]. Previously, diagnosis of clinical PCME was made based on reduced visual acuity assessment with an incidence of only 1-2%, requiring angiographic studies to support the diagnosis [4]. The occurrence of angiographic cystoid macular edema (CME) one to two months postoperatively ranges from 20% to 30% [5]. The incidence of PCME observed on OCT following contemporary phacoemulsification may vary from 4% to 11% [6], with subtle macular changes occurring in up to 41% of cases [7]. OCT macula serves as a quick and excellent tool for diagnosis and monitoring due to its minimal invasiveness.

The pathophysiology of PCME is likely complex, with inflammation resulting from surgical manipulation identified as the major cause. Inflammatory mediators such as prostaglandins, vascular endothelial growth factor, and insulin-like growth factor-1 disrupt the blood-aqueous and blood-retinal barriers, resulting in increased vascular permeability [8,9]. PCME is characterized by the accumulation of transudates in the outer plexiform layer (OPL) and inner nuclear layers (INL) of the retina, leading to cystic spaces within the retinal layers. CME begins with INL changes, progresses to combined INL and OPL morphology, and may continue to form a subretinal fluid and subsequently lead to reduced central vision [10].

Predisposing surgical risk factors for the development of PCME include posterior capsular rent with or without vitreous loss, iris trauma, retained lens fragment, usage of iris-claw or anterior chamber intraocular lens (ACIOL), and early postoperative YAG capsulotomy [2]. Non-surgical risk factors include age, male sex, retinal vein occlusion (RVO), presence of epiretinal membrane, usage of topical prostaglandin analogs, pre-existing diabetic maculopathy, and uveitis [1,11]. 

PCME is a commonly self-limiting condition; chronic PCME, which persists for more than three months, might impair vision and can be challenging to treat. The primary treatment is the usage of topical nonsteroidal anti-inflammatory medications (NSAIDs), utilized either alone or in conjunction with topical corticosteroids [12]. Alternative therapies for recalcitrant PCME include subtenons or intravitreal corticosteroid injections to inhibit arachidonic acid release, which is the precursor of the formation of prostaglandins, leukotrienes, and thromboxanes [5]. Both dexamethasone (DEX) implants and intravitreal triamcinolone acetonide (IVTA) are effective in restoring visual function and reversing morphological changes in individuals with PCME for a minimum of six months; however, the IVTA group required recurrent intravitreal injections. DEX implants are well-tolerated with a lower incidence of ocular hypertension as compared to IVTA, hence making it a favorable treatment option in PCME [13]. Intravitreal anti-vascular endothelial growth factor drugs may be reserved for individuals who are unresponsive to steroid therapy, at risk of elevated intraocular pressure, and have concomitant macular degeneration. Prophylactic interventions such as NSAIDs alone or in conjunction with steroids reveal their efficacy in lowering the incidence of PCME based on a systematic review and meta-analysis [14].

The lack of randomized controlled trials or standardized treatment protocols for PCME has made it challenging for clinicians to determine the most effective management strategies. A stepwise treatment approach was used in our case of PCME, which did not respond to the initial treatment of NSAIDs. The optimal response observed with sustained-release intravitreal 0.7mg dexamethasone implant in our patient for a longstanding PCME provides encouraging results [15]. Larger clinical trials and comparative studies are essential to establish a more comprehensive understanding of the condition and help identify effective treatment strategies to improve patient outcomes.

## Case presentation

A 58-year-old healthy Chinese male without underlying systemic comorbidities presented with bilateral cloudy vision six weeks post cataract surgery. He complains of difficulty in navigating on the roads as an e-hailing driver. He underwent an uneventful phacoemulsification for his left eye followed by his right eye, an interval of one week apart. Both eyes received a single-piece acrylic hydrophobic lens (Hoya VivinexTM XC1) (Hoya Corporation, Japan) into the capsular bags by an experienced surgeon. Intracameral adjuncts were not used in these eyes. The patient received postoperative topical dexamethasone 0.1% and ciprofloxacin 0.3% every two hours for the first week, followed by a weekly weaning regimen in the next four weeks. His past ocular history consists of being highly myopic with bilateral power of -8.50D and also having had an inferior retinal break of the right eye where a barricade laser was done to seal the break 14 months ago.

**Figure 1:**
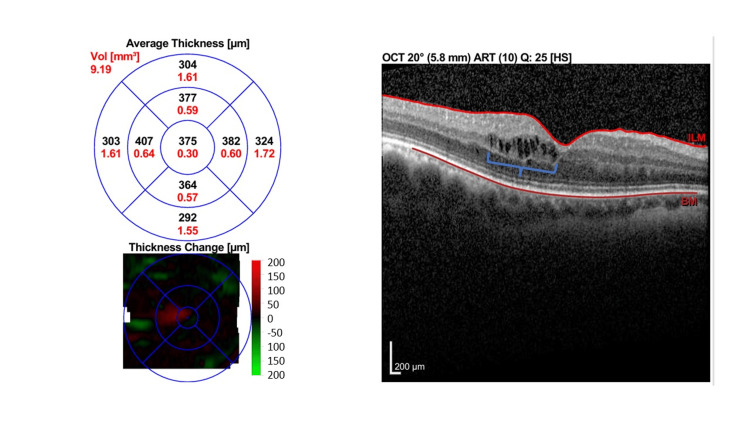
Macular OCT of right eye at six weeks postoperative. Macular OCT of the right eye (OD) showed intraretinal cystic fluid spaces (blue brace) with CST of 375 µm. OCT: Optical coherence tomography

**Figure 2:**
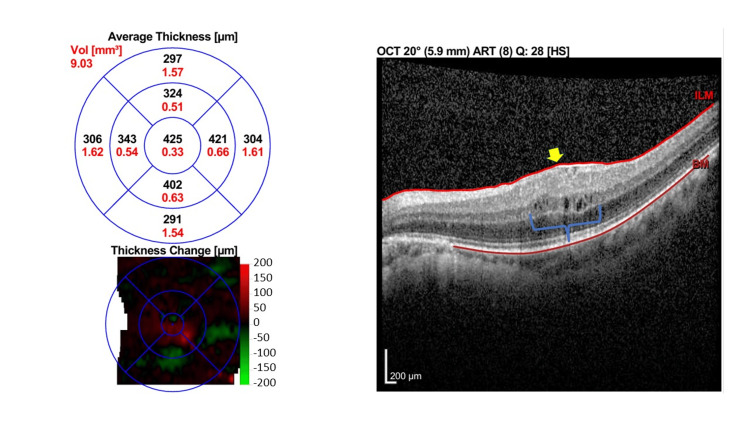
Macular OCT of left eye at six weeks postoperative. Macular OCT of the left eye (OS) showed few intraretinal cystic fluid spaces (blue brace) with loss of foveal pit contour (yellow arrow) due to the presence of epiretinal membrane with CST of 425 μm. OCT: Optical coherence tomography

His best corrected visual acuity (BCVA) measured with the Snellen chart was 6/15 OD and 6/18 OS, which was initially thought to be attributed to bilateral early-onset posterior capsular opacity (PCO). His immediate postoperative BCVA via ETDRS chart was 6/9 in both eyes (OU) with normal fundus findings. At six weeks postoperative follow-up, optical coherence tomography (OCT) revealed bilateral intraretinal cystic lesions indicating cystoid macular edema with the epiretinal membrane of the left eye with the central subfoveal thickness (CST) of 375 µm OD and 425 µm OS, respectively (Figures 1, 2).

Upon examination, bilateral anterior segment examination was unremarkable with stable intraocular lenses placed in the capsular bags. A bilateral fundus examination revealed dull foveal reflexes and retinal thickening with normal optic discs and a tessellated appearance of the retina with previous barricade laser scars over the inferonasal retina of his right eye. There was no retained cortical matter nor any signs of uveitis seen. A diagnosis of bilateral Irvine-Gass Syndrome (IGS) was made, and the patient was initiated on topical nepafenac 0.1% (Novartis Pharmaceuticals, Switzerland) three times daily for three months. Blood investigations included glycemic index, full blood count, renal profile, and infectious and inflammatory markers were all normal. His blood pressure was in the normotensive range. Bilateral posterior capsulotomy was performed by using a neodymium-doped yttrium aluminum garnet (Nd:YAG) laser six months after cataract surgery.

**Figure 3:**
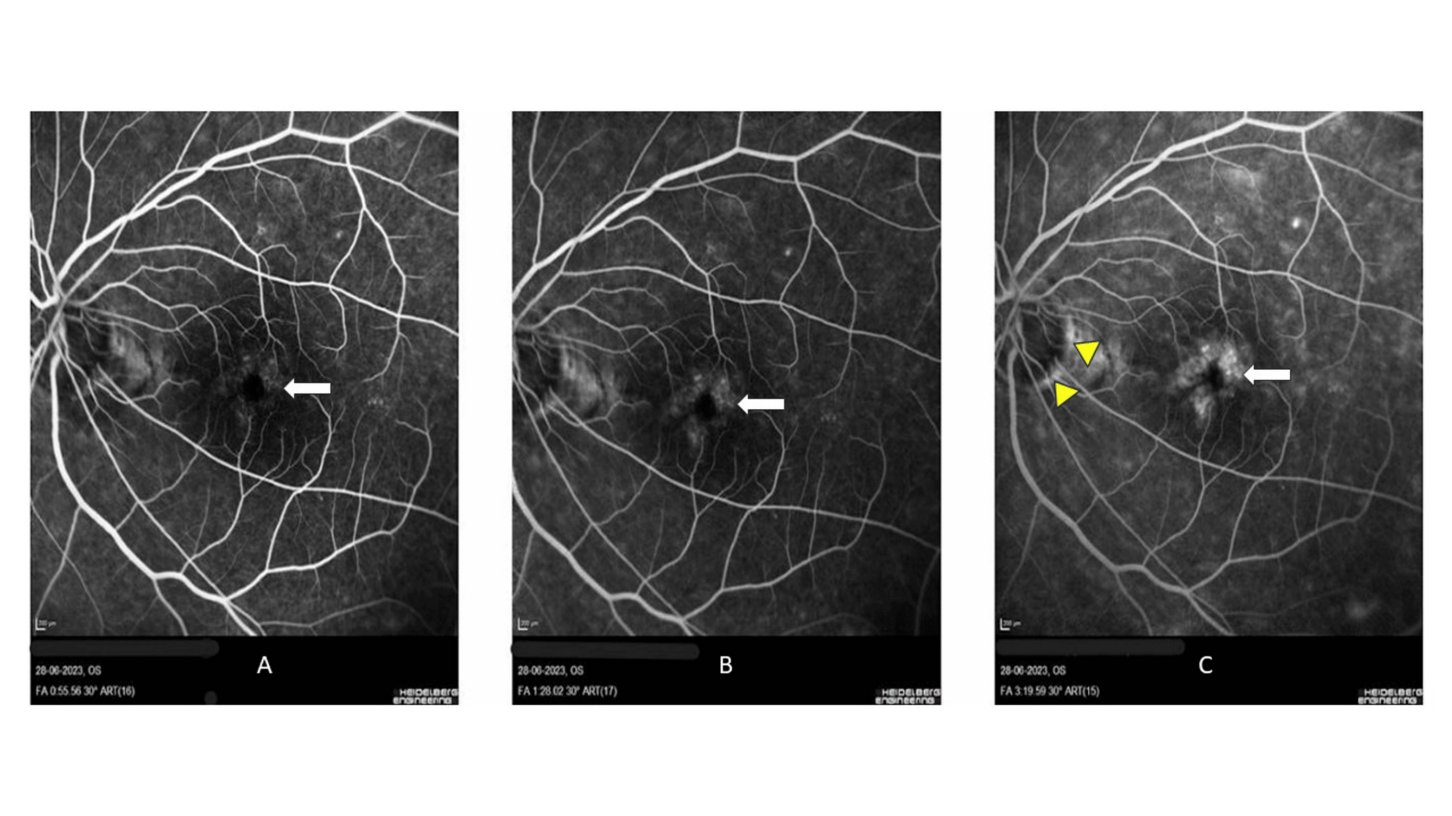
Fundus fluorescein angiography (FFA). Fundus fluorescein angiography (FFA) shows leakage of perifoveal capillaries in the early frame (white arrows) (A), increasing in size and intensity (white arrows) (B), which progresses to petaloid pattern leakage at the macula (white arrows) with staining of the temporal region optic disc (short yellow arrows) in the late frame (C).

Unfortunately, there was only mild improvement in BCVA to 6/12 OU, and persistent cystoid macular edema was seen on OCT despite three months of usage of topical nepafenac. Hence, fundus fluorescein angiography (FFA) was performed to look for other potential causes of cystoid macular edema. It revealed hyperfluorescent petaloid pattern leakage with staining of the optic disc, which correlates with findings of pseudophakic cystoid macular edema (Figure 3). There was no hot disc or any leakage from the vessels to suggest uveitis.

**Figure 4:**
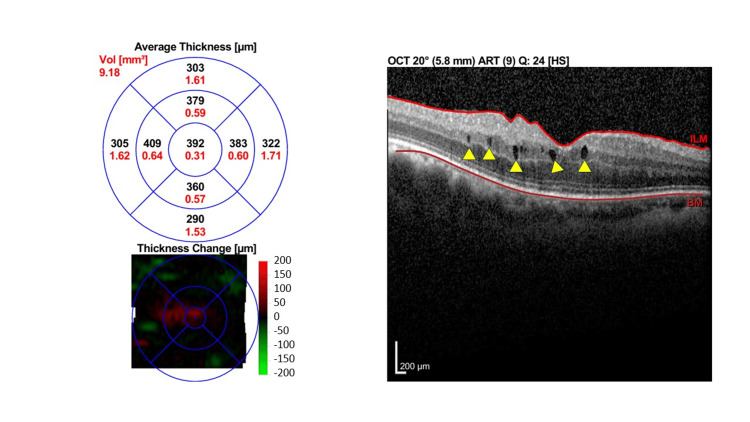
Macular OCT of right eye one month post-orbital floor triamcinolone injection. One month post orbital floor triamcinolone injection with persistent intraretinal fluid (yellow arrows) and increasing CST of 392 μm. OCT: Optical coherence tomography

**Figure 5:**
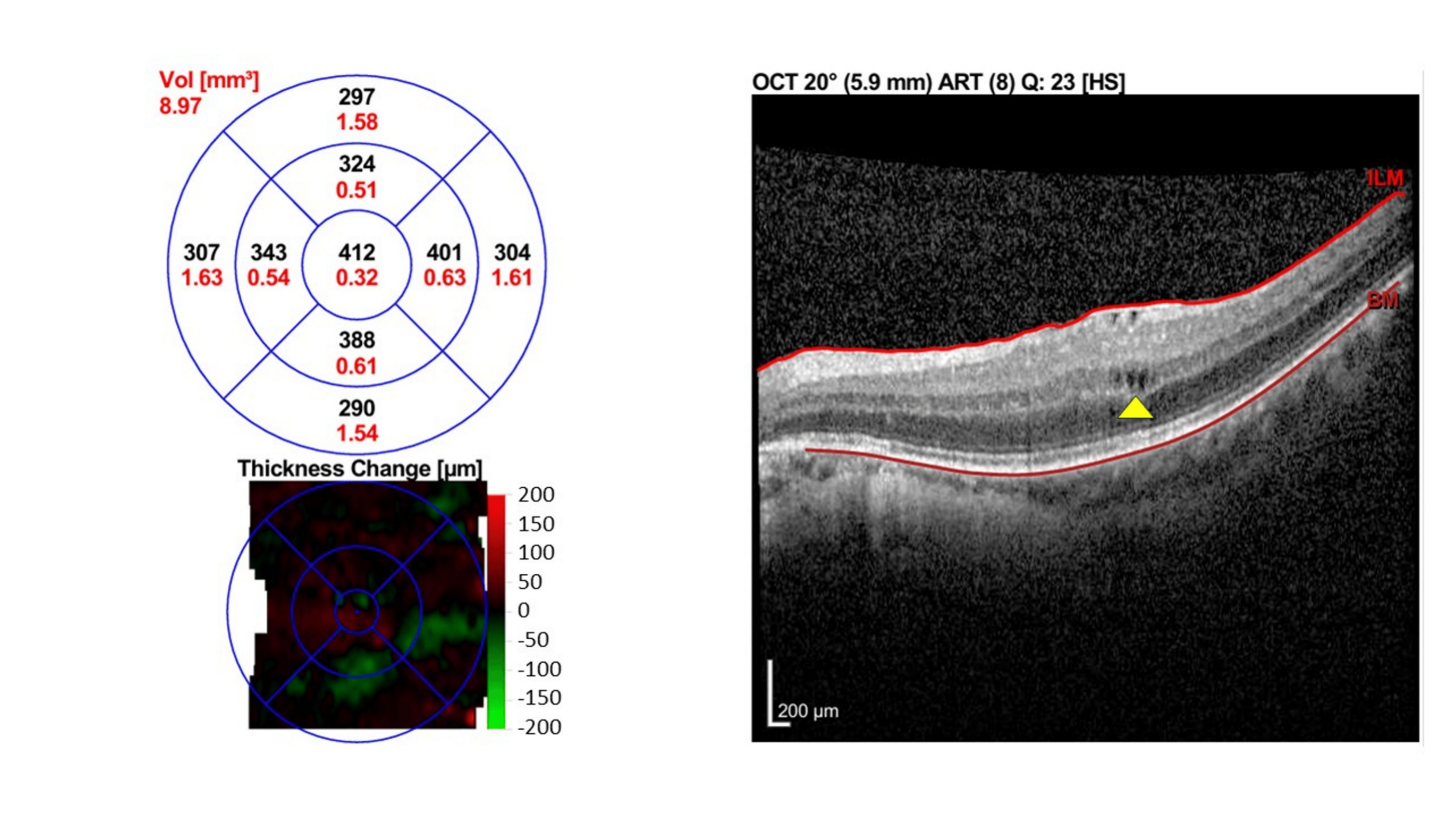
Macular OCT of left eye one month post orbital floor triamcinolone injection. Macular OCT one month post orbital floor triamcinolone injection showing few intraretinal cystic spaces (yellow arrow) with a slightly reduced CST of 412 µm. OCT: Optical coherence tomography

Due to financial reasons and inability to afford anti-vascular endothelial growth factor (VEGF) treatment, the patient was treated with orbital floor triamcinolone injection (OFTA) 40 mg/1 ml. One-month post-OFTA, BCVA improved slightly to 6/12 OU. OCT macula showed only minimal improvement with persistent intraretinal fluid in both eyes. There was an increase in CST in the right eye to 392 µm and a slight reduction of CST in the left eye to 412µm (Figure 4 [OD] and Figure 5 [OS]). 

**Figure 6:**
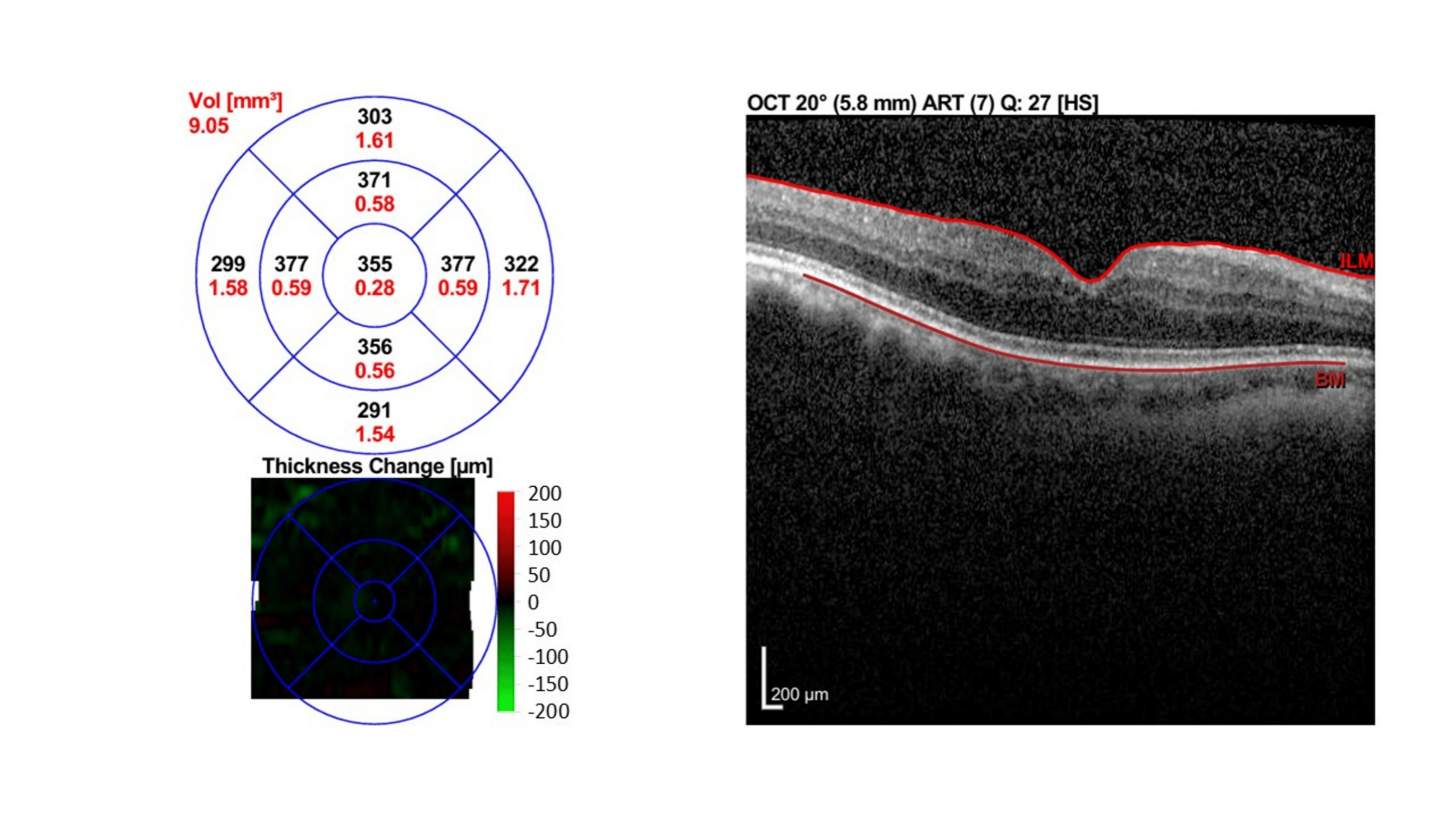
Macular OCT of the right eye one month post sustained-release intravitreal dexamethasone implant. Macular OCT shows resolution of PCME one month after treatment with sustained-release intravitreal dexamethasone implant with CST of 355 µm. OCT: Optical coherence tomography

**Figure 7:**
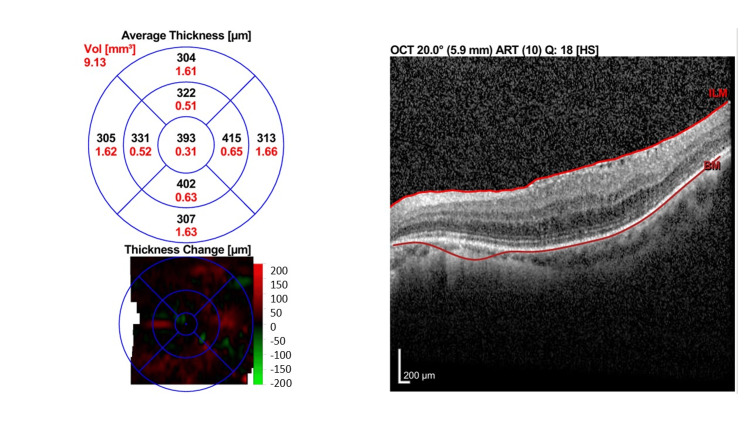
Macular OCT of the left eye one month after sustained-release intravitreal dexamethasone implant. There was resolution of PCME one month after the treatment with sustained-release intravitreal dexamethasone implant, where CST was reduced to 393 μm. OCT: Optical coherence tomography

Treatment was further escalated to sustained-release intravitreal dexamethasone implant 0.7 mg in both eyes, where it led to resolution of bilateral macular edema with BCVA 6/9 OU and was maintained throughout 120 days (Figure 6 [OD] and Figure 7 [OS]). 

## Discussion

Irvine-Gass syndrome (IGS), also known as pseudophakic cystoid macular edema, was initially discovered by ophthalmologist A. Ray Irvine Jr., MD, in 1953 after mysteriously observing it in some of his post-cataract-removal patients [16]. The condition was further evaluated by fluorescein angiography in 1969 by Gass JD and Norton EW [17]. PCME remains a common cause of postoperative reduced vision following both complicated and uncomplicated cataract surgery; therefore, it continues to be a prevalent morbidity due to the immense quantities of cataract surgeries performed.

The incidence of clinically significant PCME is about 0.1-2.4% in the phacoemulsification group [1], but detection by OCT of the macula increases up to 4-11% [6]. IGS is further classified based on duration of presentation: acute within six months and chronic if more than six months. The nature of this disease is self-limiting and has spontaneous resolution within 3 to 12 months [18]. However, few cases of prolonged and chronic PCME have been reported causing permanent visual impairment.

Complex cataract surgeries with vitreous loss, vitreous traction at the incision site, posterior capsular rupture, retained lens fragment, and iris trauma are risk factors associated with the development of PCME. Other factors contributing to IGS are prior history of diabetic macular edema, uveitis, usage of topical antiglaucoma drops, specifically latanoprost and timolol, history of retinal vein occlusion (RVO), and presence of epiretinal membrane (ERM) [19]. Intraocular lens (IOL) selection, such as iris-fixated IOLs, has a higher chances of development of IGS as compared to posterior chamber IOLs [2].

Another predisposing factor for the development of CME in this patient is the YAG posterior capsulotomy laser, which he underwent for bilateral posterior capsular opacity. There is a 0.5%-2.5% chance of developing cystoid macular edema following the YAG capsulotomy laser, which peaks between three weeks and eleven months after the procedure [20]. A longer interval between the post-cataract extraction period and laser capsulotomy is recommended to minimize the risk of CME. Our patient underwent bilateral Nd:YAG capsulotomy laser six months after the cataract surgeries, which was the recommended time.

The pathophysiology behind the development of IGS is multifactorial and based on experimental research and clinical observations. The primary mechanism is most likely due to anterior segment inflammation induced surgically, causing endogenous inflammatory mediators such as prostaglandins, cytokines, and other vasopermeability factors to be released, which leads to fluid accumulation as a result of disruption of the perifoveal retinal capillaries by these factors. Apart from prostaglandin, other chemical factors such as vascular endothelial growth factor and insulin-like growth factor-1 are further released into the vitreous when there is surgical damage to the iris, ciliary body, and lens epithelium due to compromise in the blood-aqueous barrier (BAB).

Slit lamp biomicroscopy could help to identify features of clinical PCME such as intraretinal parafoveal cystic changes, loss of foveal contour, and retinal thickening. Non-invasive imaging modalities such as spectral domain OCT macula are able to demonstrate cystic fluid spaces in both outer nuclear and outer plexiform layers. OCT macula is an important and sensitive tool for diagnostic and monitoring purposes. Fundus fluorescein angiography (FFA) is the gold standard in diagnosing IGS and is able to rule out other causes of CMO, such as diabetic macular edema or RVO [19]. Early-phase FFA reveals hyperfluorescent perifoveal capillary leakage, which transforms into a ‘petaloid’ pattern during the late phase of FFA. Petaloid fluorescein accumulation around the fovea with staining of the optic disc is a typical characteristic of PCME. Approximately 20% of patients who undergo uncomplicated phacoemulsification will present with angiographic CME [21].

There is currently no established protocol for the management and prophylaxis of PCME due to a lack of strong randomized clinical trials and studies. The management tailored for this condition is based on the pathogenesis of the disease, which targets the inflammatory cascade causing the edema. Off-label usage of topical non-steroidal anti-inflammatory drugs (NSAIDs) is both the mainstay treatment and prevention of IGS. NSAIDs are potent inhibitors of prostaglandin, where they competitively inhibit the cyclooxygenase (COX) isoforms COX-1 and COX-2, thus preventing the conversion of arachidonic acid from the cell membrane phospholipids into prostaglandin. COX-2 is a protein variant predominantly found in retinal pigmented epithelium. Topical NSAIDs that are FDA-approved for postoperative inflammation are nepafenac 0.1%, ketorolac 0.5%, and bromfenac 0.09%. In a retrospective study of 450 patients conducted by Wolf et al., they found that five patients in the group not receiving nepafenac medication developed PCME (p =.0354) compared to those who received prophylactic treatment with both nepafenac 0.1% in addition to prednisolone eye drops, who had no incidences of clinical PCME [22]. Wittpenn et al. demonstrated that clinical PCME developed in 5 out of 278 patients who were given prophylactic topical prednisolone, while no patients developed PCME under the ketorolac (p =.032) group in a multicenter masked randomized clinical trial [23]. The Miyake Hospital Eye Study carried out a randomized, double-masked, single-center clinical trial comparing the non-steroidal anti-inflammatory drug nepafenac 0.1% and the steroidal anti-inflammatory drug fluorometholone (FML) 0.1%; the former had better efficacy than the latter mentioned drug in preventing PCME and BAB disruption [24]. Ocular side effects of NSAIDs (1-5%) include lid margin crusting, conjunctival hyperaemia and allergy, punctate epithelial erosions, corneal edema, and even corneal melt in serious adverse events [25].

Corticosteroids play a role in suppressing the formation of prostaglandin and leukotriene by inhibiting phospholipase A2 in the arachidonic acid cascade. Its ability to prevent macrophage and neutrophil migration reduces vascular permeability and vasodilation [26]. Heier JS et al., 2000, explored the option of combination therapy by evaluating the efficacy of administering ketorolac 0.5% and prednisolone acetate 1.0% ophthalmic solution together for IGS [27]. Groups with combination therapy achieved two lines of more gain in visual acuity compared to either monotherapy group, most likely due to synergistic effects of drugs.

PCMEs that do not improve with topical treatment may consider the usage of periocular and intraocular corticosteroid injections. Ocular injection sites such as sub tenon, subconjunctival, and orbital floor are relatively safe and carry small risks of globe perforation. The duration of drug action may last up to three months, but the maximal efficacy of corticosteroids requires up to six weeks to take effect. In a group of 48 patients with refractory PCME, Thach et al. showed that there was no statistically significant difference in the outcomes between the two methods for sub-Tenon's injections, which improved visual acuity from 20/92 to 20/50 (p=0.0001), and retrobulbar injections, which improved visual acuity from 20/97 to 20/58 (p=0.035) [28]. A small retrospective investigation of six eyes by Suleman H et al. revealed significant reductions in retinal thickness along with visual improvement after receiving an injection of 40 mg of OFTA [29].

Benhamou et al. (2003) first reported the usage of 8 mg intravitreal triamcinolone to treat three patients with refractory chronic PCME. The study demonstrated only transient improvements in macular thickness and visual acuity [30]. One of the first controlled studies was recently published by Ahmadabadi et al., which involved 41 diabetic eyes with moderate non-proliferative diabetic retinopathy that were randomized to receive intravitreal triamcinolone at the end of the case of routine phacoemulsification. The results showed that 0 eyes in the treatment group and 4 eyes in the routine treatment group developed pseudophakic CME (p =.059). At one month, the treatment group's improvements in visual acuity were greater (p=.045), although the visual gains were not statistically significant at six months, which proved the transient effects of IVTA, which needed repetitive injections [31]. 

Sustained-release intravitreal implants, e.g., Ozurdex (AbbVie [Allergan], Illinois, USA), with potent preservative-free dexamethasone are delivered via injectable, biodegradable intravitreal drug-delivery systems (DDS) and are approved for usage in diabetic macular edema, CMO following retinal vein occlusions, and non-infectious posterior uveitis [32]. In a phase II dexamethasone DDS multicenter study conducted by Williams et al., a subset of 41 patients with persistent cystoid macular edema (PCMO) (> or = 90 days) were randomly assigned to receive either observation or 350 micrograms or 700 micrograms of dexamethasone via DDS. At day 90, 41.7% (5/12) of patients in the 350-microgram group, 53.8% (7/13) of patients in the 700-microgram group, and 14.3% (2/14) of patients in the observation group all showed a 10-letter or greater BCVA improvement (p =.029 vs. the 700-microgram group) [33]. Seven individuals (p =.079) had increased intraocular pressure, which was treated with either topical antiglaucoma or close observation. Visual acuity continued to improve until day 180. In 2019, a novel form of fluocinolone acetonide micro-insert was unveiled (Yutiq, EyePoint Pharmaceuticals, Watertown, MA) [34]. It has achieved FDA approval for non-infectious posterior uveitis and is expected to be an alternative therapeutic option for those with recalcitrant IGS. Common side effects of corticosteroids are cataracts, steroid-induced glaucoma, endophthalmitis, and inflammation.

Vascular endothelial growth factor (VEGF) plays a role in the breakdown of the blood-retinal barrier, thus causing an increase in vascular permeability leading to edema. In a subsequent study by Arevalo et al., who examined a group of 36 eyes that had chronic IGS and were unresponsive to topical and periocular injections, intravitreal bevacizumab (Avastin, Genentech, California, USA) has demonstrated efficacy in refractory cases [35]. They found that 26 eyes (72%) had at least two ETDRS lines of BCVA improvement and that at 12 months, mean baseline BCVA and central macular thickness had improved from 20/200 and 500 microns, respectively, to 20/80 and 286 microns, respectively, following a mean of 2.7 injections per eye (p < 0.0001). Intravitreal bevacizumab 1.25 mg appears to have a good safety profile in another 10-patient study by Barone et al. in treating refractory PCME by improving both BCVA and reducing central macular thickness (CMT) [36]. 

When medical therapy is unsuccessful in improving PCME, surgical intervention such as pars plana vitrectomy should be considered, particularly in cases of PCME with vitreomacular traction. Vitrectomy is targeted to remove the inflammatory mediators in the vitreous that are responsible for the pathogenesis of PCME. A five-year prospective, randomized, controlled, collaborative study carried out by Fung et al. in 1985 discovered that eyes that had vitrectomies had superior visual outcomes compared to controls [37]. Another study by Pendergast et al. in 1999 proved that all 23 patients with refractory IGS without any vitreous abnormalities were subjected to vitrectomy; some were able to have resolution of PCME [38]. The role of vitrectomy in PCME should be further evaluated in future studies.

## Conclusions

Despite our modern-world cataract surgery with the evolution of technique and precision, PCME remains one of the most common postoperative complications even in an uneventful surgery or any prior risk factors. Surgical manipulation might contribute to the inflammatory-driven pathogenesis, releasing mediators such as prostaglandins, arachidonic acid, cytokines, lysozymes, and vascular endothelial growth, leading to disruption of the blood-aqueous and blood-retinal barriers, hence increasing vascular permeability leading to edema. The vast majority of cases will be resolved without intervention. Management of recalcitrant PCMEs is challenging due to the lack of distinct guidelines or consensus regarding the stepwise treatment approach. We emphasize and summarize the diverse approach to managing PCME in patients who have failed initial therapies. Current literature is in favor of the use of short-acting intravitreal corticosteroids, sustained-release corticosteroid implants, and intravitreal anti-VEGF agents for the treatment of refractory PCME. Larger clinical trials are needed to provide reliable guidelines and treatment protocols. These trials will help establish more definitive treatment and prevent permanent visual morbidity in our patients.
